# Polynomial Identification of $\omega$-Automata

**DOI:** 10.1007/978-3-030-45237-7_20

**Published:** 2020-03-13

**Authors:** Dana Angluin, Dana Fisman, Yaara Shoval

**Affiliations:** 8 Johannes Kepler University, Linz, Austria; 9 University of Birmingham, Birmingham, UK; 10 Yale University, New Haven, CT USA; 11 Ben-Gurion University, Be’er Sheva, Israel

**Keywords:** identification in the limit, characteristic sample, $\omega$-regular

## Abstract

We study identification in the limit using polynomial time and data for models of $\omega$-automata. On the negative side we show that non-deterministic $\omega$-automata (of types Büchi, coBüchi, Parity or Muller) can not be polynomially learned in the limit. On the positive side we show that the $\omega$-language classes $\mathbb {IB}$, $\mathbb {IC}$, $\mathbb {IP}$, and $\mathbb {IM}$ that are defined by deterministic Büchi, coBüchi, parity, and Muller acceptors that are isomorphic to their right-congruence automata (that is, the right congruences of languages in these classes are fully informative) are identifiable in the limit using polynomial time and data. We further show that for these classes a characteristic sample can be constructed in polynomial time.

## 1 Introduction

With the growing success of machine learning in efficiently solving a wide spectrum of problems, we are witnessing an increased use of machine learning techniques in formal methods for system design. One thread in recent literature uses general purpose machine learning techniques for obtaining more efficient verification/synthesis algorithms. Another thread, following the *automata theoretic approach to verification* [33,21] works on developing grammatical inference algorithms for verification and synthesis purposes. *Grammatical inference* (aka *automata learning*) refers to the problem of automatically inferring from examples a finite representation (e.g. an automaton, a grammar, or a formula) for an unknown language. The term *model learning* [31] was coined for the task of learning an automaton model for an unknown system. A large body of works has developed learning techniques for different automata types (e.g. I/O automata [1], register automata [20], symbolic automata [14], $\omega$-automata [7], and program automata [25]) and has shown its usability in a diverse range of tasks.[3]

In grammatical inference, the learning algorithm does not learn a *language*, but rather a finite *representation* of it. Complexity of learning algorithms may

* This research was supported by grant 2016239 from the United States – Israel Binational Science Foundation (BSF).

[3] E.g., tasks such as black-box checking [28], specification mining [2], assume-guarantee reasoning [13], regular model checking [18], learning verification fixed-points [32], learning interfaces [27], analyzing botnet protocols [12] or smart card readers [10], finding security bugs [10], error localization [11], and code refactoring [26,29].

vary greatly by switching representations. For instance, if one wishes to learn regular languages, she may consider representations using deterministic finite automata (DFAs), non-deterministic finite automata (NFAs), regular expressions, linear grammars etc. Since the translation results between two such formalisms are not necessarily polynomial, a polynomial learnability result for one representation does not necessarily imply a polynomial learnability result for another representation. Let $\mathbb{C}$ be a class of representations $\mathcal{C}$ with a size measure $size(\mathcal{C})$ (e.g. for DFAs the size measure can be the number of states in the minimal automaton). We extend $size(\cdot)$ to the languages recognized by representations in $\mathbb{C}$ by defining $size(L)$ to be the minimum of $size(\mathcal{C})$ over all $\mathcal{C}$ representing $L$. In this paper we restrict attention to automata representations, namely, *acceptors*.

There are various learning paradigms considered in the grammatical inference literature, roughly classified into *passive* and *active*. We mention here the two central ones. In *passive learning* the model of *learning from finite data* refers to the following problem: given a finite sample $T \subseteq \Sigma^* \times \{0,1\}$ of labeled words, a learning algorithm $\mathbf{A}$ should return an acceptor $\mathcal{C}$ that agrees with the sample $T$. That is, for every $(w, l) \in T$ the following holds: $w \in [\![\mathcal{C}]\!]$ iff $l = 1$ (where $[\![\mathcal{C}]\!]$ is the language accepted by $\mathcal{C}$). The class $\mathbb{C}$ is *identifiable in the limit using polynomial time and data* if and only if there exists a polynomial time algorithm $\mathbf{A}$ that takes as input a labeled sample $T$ and outputs an acceptor $\mathcal{C} \in \mathbb{C}$ that is consistent with $T$, and $\mathbf{A}$ also satisfies the following condition. If $L$ is any language recognized by an automaton from class $\mathbb{C}$, then there exists a labeled sample $T_L$ consistent with $L$ of length bounded by a polynomial in $size(L)$, and for any labeled sample $T$ consistent with $L$ such that $T_L \subseteq T$, on input $T$ the algorithm $\mathbf{A}$ produces an acceptor $\mathcal{C}$ that recognizes $L$. In this case, $T_L$ is termed a *characteristic sample* for the algorithm $\mathbf{A}$. In some cases (e.g., DFAs) there is also a polynomial time algorithm to compute a characteristic sample for $\mathbf{A}$, given an acceptor $\mathcal{C} \in \mathbb{C}$.

In *active learning* the model of *query learning* [5] assumes the learner communicates with an *oracle* (sometimes called *teacher*) that can answer certain types of queries about the language. The most common type of queries are *membership queries* (is $w \in L$ where $L$ is the unknown language) and *equivalence queries* (is $[\![\mathcal{A}]\!] = L$ where $\mathcal{A}$ is the current hypothesis for an acceptor recognizing $L$). Equivalence queries are typically assumed to return a counterexample, i.e. a word in $[\![\mathcal{A}]\!] \setminus L$ or in $L \setminus [\![\mathcal{A}]\!]$.

With regard to $\omega$-automata (automata on infinite words) most of the works consider *query learning*. The representations learned so far include: $(L)_\$$ [15], a non-polynomial reduction to finite words; families of DFAs ($\mathbb{FDFA}$) [7,8,6,22]; strongly unambiguous Büchi automata ($\mathbb{SUBA}$) [3]; and deterministic weak parity automata ($\mathbb{DWPA}$) [23]. Among these only the latter is learnable in polynomial time using membership queries and proper equivalence queries.

One of the main obstacles in obtaining a polynomial learning algorithm for $\omega$-regular languages is that they do not in general have a Myhill-Nerode characterization; that is, there is no theorem correlating the states of a minimal automaton of some of the common automata types (Büchi, Parity, Muller, etc.)

to the equivalence classes of the right congruence of the language. The *right congruence relation* for an $\omega$-language $L$ relates two finite words $x$ and $y$ iff there is no infinite suffix $z$ differentiating them, that is $x \sim_L y$ (for $x, y \in \Sigma^*$) iff $\forall z \in \Sigma^\omega.\ xz \in L \iff yz \in L$. In our quest for finding a polynomial query learning algorithm for a subclass of the $\omega$-regular languages, we have studied subclasses of languages for which such a relation holds [4], and termed them *fully informative*. We use $\mathbb{IB}, \mathbb{IC}, \mathbb{IP}, \mathbb{IM}$ to denote the classes of languages that are fully informative of type Büchi, coBüchi, Parity and Muller, respectively. A language $L$ is said to be fully informative of type $\mathbb{X}$ for $\mathbb{X} \in \{\mathbb{B}, \mathbb{C}, \mathbb{P}, \mathbb{M}\}$ if there exists a deterministic automaton of type $\mathbb{X}$ which is isomorphic to the automaton derived from $\sim_L$. While a lot of properties about these classes are now known, in particular that they span the entire hierarchy of $\omega$-regular properties [34], a polynomial learning algorithm for them has not been found yet.

In this paper we show that the classes $\mathbb{IB}, \mathbb{IC}, \mathbb{IP}, \mathbb{IM}$ can be identified in the limit using polynomial time and data. We further show that there is a polynomial time algorithm to compute a characteristic sample given an acceptor $\mathcal{C} \in \mathbb{IX}$. A corollary of this result is that the class of languages accepted by $\mathbb{DWPA}$s (which as mentioned above is polynomially learnable in the query learning setting) also has a polynomial size characteristic sample. On the negative side, we show that the classes $\mathbb{NBA}$, $\mathbb{NCA}$, $\mathbb{NPA}$, $\mathbb{NMA}$ of non-deterministic Büchi, coBüchi, Parity and Muller automata, resp., cannot be identified in the limit using polynomial data.

## 2 Preliminaries

*Automata and Acceptors* An *automaton* is a tuple $\mathcal{A} = \langle \Sigma, Q, q_\iota, \delta \rangle$ consisting of a finite totally ordered alphabet $\Sigma$ of symbols, a finite set $Q$ of states, an initial state $q_\iota \in Q$, and a transition function $\delta : Q \times \Sigma \to 2^Q$. A run of an automaton on a finite word $v = a_1 a_2 \ldots a_n$ is a sequence of states $q_0, q_1, \ldots, q_n$ such that $q_0 = q_\iota$, and for each $i \geq 0$, $q_{i+1} \in \delta(q_i, a_{i+1})$. A run on an infinite word is defined similarly and results in an infinite sequence of states. We say that $\mathcal{A}$ is *deterministic* if $|\delta(q, a)| \leq 1$ and *complete* if $|\delta(q, a)| \geq 1$, for every $q \in Q$ and $a \in \Sigma$. We extend $\delta$ to domain $Q \times \Sigma^*$ in the usual manner, and abbreviate $\delta(q, \sigma) = \{q'\}$ as $\delta(q, \sigma) = q'$.

By augmenting an automaton with an acceptance condition $\alpha$, obtaining a tuple $\langle \Sigma, Q, q_\iota, \delta, \alpha \rangle$, we get an *acceptor*, a machine that accepts some words and rejects others. An acceptor accepts a word if at least one of the runs on that word is accepting. For finite words the acceptance condition is a set $F \subseteq Q$ and a run on a word $v$ is accepting if it ends in an accepting state, i.e., if $\delta(q_\iota, v)$ contains an element of $F$. For infinite words, there are various acceptance conditions in the literature; we consider four: Büchi, coBüchi, parity, and Muller. The Büchi and coBüchi acceptance conditions are also a set $F \subseteq Q$. A run of a Büchi automaton is accepting if it visits $F$ infinitely often. A run of a coBüchi is accepting if it visits $F$ only finitely many times. A parity acceptance condition is a map $\kappa : Q \to \mathbb{N}$ assigning each state a natural number termed a color (or priority). A run is accepting if the **minimum** color visited infinitely often is **odd**.

A Muller acceptance condition is a set of sets of states $\alpha = \{F_1, F_2, \ldots, F_k\}$ for some $k \in \mathbb{N}$ and $F_i \subseteq Q$ for $i \in [1..k]$. A run of a Muller automaton is accepting if the set $S$ of states visited infinitely often in the run is a member of $\alpha$. We use $[\![\mathcal{A}]\!]$ to denote the set of words accepted by a given acceptor $\mathcal{A}$. We use NBA, NPA, NMA, NCA for non-determinstic Büchi, parity, Muller and coBüchi, automata. We use $\mathbb{NBA}$, $\mathbb{NPA}$, $\mathbb{NMA}$ and $\mathbb{NCA}$ for the classes of languages they recognize. The first three recognize the full class of $\omega$-regular languages while the forth only a subset of it.

*Right congruences* An equivalence relation $\sim$ on $\Sigma^*$ is a *right congruence* if $x \sim y$ implies $xv \sim yv$ for every $x, y, v \in \Sigma^*$. The *index* of $\sim$, denoted $|{\sim}|$ is the number of equivalence classes of $\sim$. Given a language $L \subseteq \Sigma^*$ its *canonical right congruence* $\sim_L$ is defined as follows: $x \sim_L y$ iff $\forall z \in \Sigma^*$ we have $xz \in L \iff yz \in L$. For a word $v \in \Sigma^*$ the notation $[v]$ is used for the equivalence class of $\sim$ in which $v$ resides.

With a right congruence $\sim$ of finite index one can naturally associate an automaton $\mathcal{M}_\sim = \langle \Sigma, Q, q_\iota, \delta \rangle$ as follows: the set of states $Q$ consists of the equivalence classes of $\sim$. The initial state $q_\iota$ is the equivalence class $[\varepsilon]$. The transition function $\delta$ is defined by $\delta([u], a) = [ua]$. Similarly, given a complete deterministic automaton $\mathcal{M} = \langle \Sigma, Q, q_\iota, \delta \rangle$ we can naturally associate with it a right congruence as follows: $x \sim_\mathcal{M} y$ iff $M$ reaches the same state when reading $x$ or $y$. The Myhill-Nerode Theorem states that a language $L$ is regular iff $\sim_L$ is of finite index. Moreover, if $L$ is accepted by a DFA $\mathcal{A}$ then $\sim_\mathcal{A}$ refines $\sim_L$. Finally, the index of $\sim_L$ gives the size of the minimal complete DFA for $L$.

For an $\omega$-language $L \subseteq \Sigma^\omega$, the right congruence $\sim_L$ is defined similarly, by quantifying over $\omega$-words. That is, $x \sim_L y$ iff $\forall z \in \Sigma^\omega$ we have $xz \in L \iff yz \in L$. Given a deterministic automaton $\mathcal{M}$ we can define $\sim_\mathcal{M}$ exactly as for finite words. However, for $\omega$-regular languages, the relation $\sim_L$ does not suffice to obtain a "Myhill-Nerode" characterization. As an example consider the language $L = (a+b)^*(bba)^\omega$. We have that $\sim_L$ consists of just one equivalence class, since for any $x \in \Sigma^*$ and $w \in \Sigma^\omega$ we have that $xw \in L$ iff $w$ has $(bba)^\omega$ as a suffix. But an $\omega$-acceptor recognizing $L$ obviously needs more than a single state.

*The classes* $\mathbb{IB}$, $\mathbb{IC}$, $\mathbb{IP}$ *and* $\mathbb{IM}$ A language $L$ is in $\mathbb{IB}$ (resp., $\mathbb{IC}$, $\mathbb{IP}$, $\mathbb{IM}$) if there exists a deterministic Büchi (resp., coBüchi, parity, Muller) acceptor $\mathcal{A}$ such that $L = [\![\mathcal{A}]\!]$ and there is a 1-to-1 relationship between the states of $\mathcal{A}$ and the equivalence classes of $\sim_L$: if $x \sim_L y$ then $x$ and $y$ reach the same state $q$ in $\mathcal{A}$, and an $\omega$-word $z$ is accepted from $q$ iff $xz \in L$ (which holds iff $yz \in L$). These classes are more expressive than one might conjecture, it was shown in [4] that in every class of the infinite Wagner hierarchy [34] there are languages in $\mathbb{IM}$ and $\mathbb{IP}$. Moreover, in a small experiment reported in [4], among randomly generated Muller automata, the vast majority turned out to be in $\mathbb{IM}$.

*Examples and samples* Since we need finite representations of examples, $\omega$-words in our case, we work with ultimately periodic words, that is, words of the form $u(v)^\omega$ where $u \in \Sigma^*$ and $v \in \Sigma^+$. It is known that two regular $\omega$-languages are equivalent iff they agree on the set of ultimately periodic words, so this choice is not limiting. The example $u(v)^\omega$ is concretely represented by the pair $(u, v)$ of finite strings, and its length is $|u| + |v|$. A *labeled example* is a pair $(u(v)^\omega, l)$, where the label $l$ is either 0 or 1. A *sample* is a finite set of labeled examples such that no example is assigned two different labels. The length of a sample is the sum of the lengths of the examples that appear in it. A sample $T$ and a language $L$ are consistent with each other if and only if for every labeled example $(u(v)^\omega, l) \in T$, $l = 1$ iff $u(v)^\omega \in L$. A sample and an acceptor are consistent with each other if and only if the sample and the language recognized by the acceptor are consistent with each other. The following results give two useful procedures on examples that are computable in polynomial time.

**Claim 1.** *Given $u_1, u_2 \in \Sigma^*$ and $v_1, v_2 \in \Sigma^+$, if $u_1(v_1)^\omega \neq u_2(v_2)^\omega$ then they differ in at least one of the first $\ell$ symbols, where $\ell = \max(|u_1|, |u_2|) + |v_1| \cdot |v_2|$.*

Let *suffixes*$(u(v)^\omega)$ denote the set of all $\omega$-words that are suffixes of $u(v)^\omega$.

**Claim 2.** *The set suffixes$(u(v)^\omega)$ consists of at most $|u|+|v|$ different examples: one of the form $u'(v)^\omega$ for every nonempty suffix $u'$ of $u$, and one of the form $(v_2v_1)^\omega$ for every division of $v$ into a non-empty prefix and suffix as $v = v_1v_2$.*

*Identification in the limit using polynomial time and data* We consider the notion of identification in the limit using polynomial time and data. This criterion of learning was introduced by [16], who showed that regular languages of finite strings represented by DFAs are learnable in this sense. We follow a more general definition given by [19]. The definition has two requirements: (1) a learning algorithm **A** that runs in polynomial time on a set of labeled examples and produces a hypothesis consistent with the examples, and (2) that for every language $L$ in the class, there exists a set $T_L$ of labeled examples of size polynomial in a measure of size of $L$ such that on any set of labeled examples containing $T_L$, the algorithm **A** outputs a hypothesis correct for $L$. Condition (1) ensures polynomial time, while condition (2) ensures polynomial data. The latter is not a worst-case measure; there could be arbitrarily large finite samples for which **A** outputs an incorrect hypothesis. However, de la Higuera shows that identifiability in the limit with polynomial time and data is closely related to a model of a learner and a helpful teacher introduced by [17].

## 3 Negative Results

We start with negative results. We show that when the representation at hand is non-deterministic, polynomial identification is not feasible.

**Theorem 3.** *The class $\mathbb{NBA}$ cannot be identified in the limit using polynomial data.*

*Proof.* The proof follows the idea given in the negative result for learning in the limit NFAs from polynomial data [19]. For any integer $M \geq 2$, let $p_1, \ldots, p_m$ be

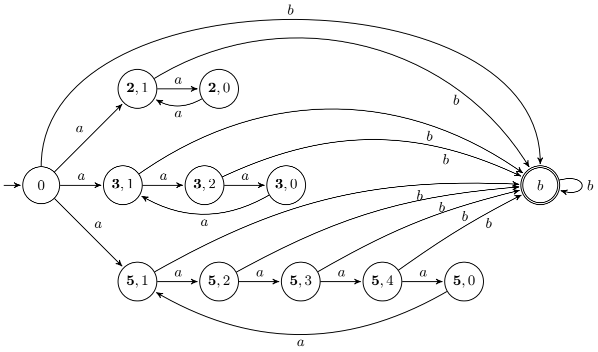

Fig. 1: The NBA $\mathcal{B}_M$ for $M = 5$.

the set of all primes less than or equal to $M$. For each such $M$, consider the NBA $\mathcal{B}_M$ over a two letter alphabet $\Sigma = \{a, b\}$ with $p_1 + p_2 + \ldots + p_m + 2$ states, where state 0 has $a$-transitions to state $(\mathbf{p}, 1)$ for each $\mathbf{p} \in \{p_1, p_2, \ldots, p_m\}$. State $(\mathbf{p}, i)$ has an $a$-transition to state $(\mathbf{p}, i \oplus_p 1)$ where $\oplus_p$ is addition modulo $p$. All states except the states $(\mathbf{p}, 0)$ have a $b$-transition to state $b$. The state $b$ has a self-loop on $b$. The only accepting state is $b$. The NBA $\mathcal{B}_M$ for $M = 5$ is given in Fig. 1.

The NBA $\mathcal{B}_M$ accepts the set of all words of the form $a^k b^\omega$ such that $k$ is *not* a positive multiple of $\ell = p_1 \cdot p_2 \cdots p_m$. Note that the size of the shortest ultimately periodic word in $a^* b^\omega \setminus [\![\mathcal{B}_M]\!]$ is $\ell + 1$, and thus, to distinguish the language $[\![\mathcal{B}_M]\!]$ from the language $a^* b^\omega$, a word of at least this size must be provided. Since the number of primes not greater than $M$ is $\Theta(M/\log M)$ and since each prime is of size at least 2 the data must be of size at least $2^{\Theta(M/\log M)}$ while the number of states of $\mathcal{B}_M$ is $O(M^2)$. □

Since NBAs are a special case of non-deterministic parity automata (NPA) and non-deterministic Muller automata (NMA) it follows that these models too cannot be identified in the limit using polynomial data. Note that indeed the NBA in the proof of Theorem 3 can be regarded as an NPA by setting the color of state $b$ to 1 and the color of all other states to 0. Likewise it can be regarded as an NMA by defining the accepting set as $\{\{b\}\}$.

**Corollary 1.** *The classes* $\mathbb{NPA}$ *and* $\mathbb{NMA}$ *cannot be identified in the limit using polynomial data.*

While NBAs are not a special case of non-deterministic coBüchi automata (NCA) it can be shown that $\mathbb{NCA}$ as well cannot be identified in the limit from polynomial data, which is in some sense surprising, since NCAs are not more expressive than DCAs, their deterministic counterpart, and accept a very small subclass of the regular $\omega$-languages.

**Theorem 4.** *The class* $\mathbb{NCA}$ *cannot be identified in the limit using polynomial data.*

*Proof.* The proof is almost identical to that of Theorem 3. The only difference is that it considers the automaton $\mathcal{C}_M$ that takes exactly the same form as $\mathcal{B}_M$ from that proof but switching accepting and non-accepting states. Since $\mathcal{C}_M$ clearly accepts the same language as that of $\mathcal{B}_M$, with the same number of states, the proof continues exactly the same. □

## 4 Outline for the positive results

The rest of the paper is devoted to the positive results. To show that a class is identified in the limit using polynomial time and data there are two steps: (i) constructing a sample of words $T_L$ of size polynomial in the given acceptor $\mathcal{M}$ for the language $L$ at hand, the so called, *characteristic sample*, and (ii) providing a learning algorithm that for every given sample $T$ returns an acceptor consistent with that sample, and in addition for any sample $T$ that subsumes $T_L$ returns an acceptor that exactly recognizes $L$.

Since the construction of the characteristic sample is simpler we start with that. We show that the classes $\mathbb{IB}$, $\mathbb{IC}$, $\mathbb{IP}$ and $\mathbb{IM}$ have characteristic samples of size polynomial in the number of states of the acceptor, and that the characteristic sample can be constructed in polynomial time. The definition of an acceptor is composed of two steps: (a) the definition of the automaton and (b) the definition of the acceptance condition. Some words are put in the sample to help retrieving the automaton and some to help retrieving the acceptance condition. We view the characteristic sample as a union of two parts $T_{Aut}$ (for retrieving the automaton) and $T_{Acc}$ (for retrieving the acceptance condition). The learning algorithm first constructs the automaton, then retrieves the acceptance condition.

In Section 5 we discuss the construction of $T_{Aut}$ which is common to all the classes we consider, as they all are isomorphic to the automaton of the right congruence. In Section 6 we show how an algorithm can retrieve the automaton using the labeled words in $T_{Aut}$. In Section 7 we discuss the construction of $T_{Acc}$ that regards the acceptance condition of the DPA. This part is the most involved one. We first associate with a DPA a canonical forest of its strongly connected components. From this canonical forest we build the $T_{Acc}$ part of the characteristic sample. In Section 8 we show a learning algorithm that can retrieve in polynomial time the acceptance condition of the DPA, from labeled examples in $T_{Acc}$. This implies that $\mathbb{IP}$ (as well as its special cases $\mathbb{IB}$ and $\mathbb{IC}$) can be learned in the limit from polynomial time and data. In Section 9 we show that the class $\mathbb{IM}$ can also be learned in the limit from polynomial time and data.

## 5 The characteristic sample for the automaton

In this section we show how to construct the $T_{Aut}$ part of the sample. We first show that any two states that are distinguishable in the automaton, are distinguishable by words of length polynomial in the number of states.

### 5.1 Polynomial construction of short distinguishing words

Let $M$ be an acceptor in one of the classes $\mathbb{IB}$, $\mathbb{IC}$, $\mathbb{IP}$ or $\mathbb{IM}$ with states $Q$ over alphabet $\Sigma$. If $M$ is in one of the first three classes we use $\max\{|\Sigma|, |Q|\}$ for its size measure. If $M \in \mathbb{IM}$ we use $\max\{|\Sigma|, |Q|, m\}$ for its size measure where $m$ is the number of sets in the acceptance condition $\alpha$. We say that states $q_1$ and $q_2$ of $\mathcal{M}$ are *distinguishable* if there exists a word $z \in \Sigma^\omega$ that is accepted from one but not the other (and that $z$ is a *distinguishing word*). First we show that any two distinguishable states of $\mathcal{M}$ are distinguishable by an ultimately periodic word of size polynomial in $\mathcal{M}$. Then we show how to use these words to construct the $T_{Aut}$ part of the characteristic sample.

**Proposition 5.** *If two states of a DMA, DPA, DBA or DCA of $n$ states are distinguishable, then they are distinguishable by an ultimately periodic $\omega$-word of length bounded by $n^2 + n^4$.*

*Proof.* We prove that for a DMA $\mathcal{M}$ of $n$ states, if two distinct states $q_1$ and $q_2$ are distinguishable, then they are distinguishable by an ultimately periodic $\omega$-word of length bounded by $n^2+n^4$. Since any DPA, DBA or DCA is equivalent to an isomorphic DMA, the above result holds also for DPAs, DBAs and DCAs.

Because $q_1$ and $q_2$ are distinguishable, there exists an ultimately periodic $\omega$-word $x(y)^\omega$ that is accepted from exactly one of the two states. For each nonnegative integer $k$ and $i = 1, 2$, let $q_i(k)$ be the state visited after $k$ symbols of $x(y)^\omega$ have been read, starting with state $q_i$. Also, let $C_i$ be the set of states visited infinitely often by the sequence $q_i(k)$, which determines the acceptance or rejection of $x(y)^\omega$ from $q_i$. The sequence of pairs $(q_1(k), q_2(k))$ for $k = 0, 1, \ldots$ takes on at most $n^2$ different values. Let $C$ be the set of pairs visited infinitely often by this sequence. The two projections $\pi_1(C)$ and $\pi_2(C)$ are $C_1$ and $C_2$.

Let $\ell$ be the minimum value for which $(q_1(k), q_2(k))$ visits only pairs in $C$ for all $k \geq \ell$. Let $x'$ be the prefix of $x(y)^\omega$ consisting of $\ell$ symbols. By removing symbols between repeated pairs $(q_1(k), q_2(k))$ from $x'$ we obtain a string $u$ of length at most $n^2$ that reaches the pair $(q_1(\ell), q_2(\ell))$ from $(q_1(0), q_2(0))$. Let $m$ be the minimum value for which $(q_1(k), q_2(k))$ for $\ell \leq k \leq m$ visits all the pairs of $C$ and returns to $(q_1(\ell), q_2(\ell))$, and let $y'$ be the string from symbol $\ell$ to $m-1$ of $x(y)^\omega$. Distinguishing a subsequence of pairs that visits each element of $C$ once, we can remove from $y'$ sequences of symbols between repeated pairs that do not include a distinguished pair between them. Thus we obtain a string $v$ of length at most $|C|n^2$, that starts at $(q_1(\ell), q_2(\ell))$, visits all the distinguished pairs and returns to the starting pair. Since $|C| \leq n^2$, the length of $u(v)^\omega$ is at most $n^2 + n^4$. Also, since the set of states visited infinitely often on input $u(v)^\omega$ from $q_i$ is $C_i$ we have that $u(v)^\omega$ is accepted from exactly one of $q_1$ and $q_2$. □

For DPAs as well as DMAs there is a polynomial time algorithm to determine whether two states are distinguishable and to find a distinguishing $\omega$-word $u(v)^\omega$ if they are. This result relies on a polynomial time algorithm to test the equivalence of two DPAs or two DMAs and return an example $u(v)^\omega$ on which they differ if not [9]. Since DBA and DCA are special cases of a DPA, a polynomial construction of a distinguishing word applies to them as well.

### 5.2 Constructing the characteristic sample for the automaton

We now show how to construct the $T_{Aut}$ part of the characteristic sample, given an acceptor $\mathcal{M}$ in one of the classes $\mathbb{IM}$, $\mathbb{IP}$, $\mathbb{IB}$ or $\mathbb{IC}$. Let $n$ be the number of states of $\mathcal{M}$. We may assume that every state of $\mathcal{M}$ is reachable from the initial state $q_\iota$. The algorithm constructs a set $S$ of $n$ *access strings* by breadth-first search in the transition graph of $\mathcal{M}$ such that $S$ is prefix-closed and contains exactly one lexicographically least string of shortest possible length reaching each state of $\mathcal{M}$ from the initial state. Using Proposition 5, the algorithm may also construct a set $E$ of at most $n^2$ distinguishing experiments that contains for each pair $q_1$ and $q_2$ of distinct states of $\mathcal{M}$, an $\omega$-word $u(v)^\omega$ of length at most $n^2 + n^4$ that is accepted from exactly one of the states $q_1$ and $q_2$.

Part one of the sample, $T_{Aut}$, consists of all the examples in $(S \cdot E) \cup (S \cdot \Sigma \cdot E)$, labeled to be consistent with $\mathcal{M}$. There are at most $(1 + |\Sigma|)n^3$ labeled examples in $T_{Aut}$, each of length bounded by a polynomial in $n$. This information is enough to allow the polynomial time learning algorithm to reconstruct a transition graph isomorphic to that of $\mathcal{M}$.

**Proposition 6.** *Let $\mathcal{M}'$ be any deterministic automaton that is consistent with the sample $T_{Aut}$. Then $\mathcal{M}'$ has at least $n$ states and if $\mathcal{M}'$ has exactly $n$ states then $\mathcal{M}'$ and $\mathcal{M}$ have isomorphic transition graphs.*

*Proof.* The states of $\mathcal{M}'$ reached from the initial state by the access strings in $S$ must all be distinct, because for any pair of different strings $s_1, s_2 \in S$, there exists a word $u(v)^\omega \in E$ such that $s_1 \cdot u(v)^\omega$ and $s_2 \cdot u(v)^\omega$ have different labels in $T_{Aut}$. Thus $\mathcal{M}'$ must have at least $n$ distinct states.

Assume that $\mathcal{M}'$ has exactly $n$ states. Given the state $q$ of $\mathcal{M}'$ reached by some $s \in S$ and a symbol $\sigma \in \Sigma$, the labeled examples $s \cdot \sigma \cdot u(v)^\omega$ in $T_{Aut}$ for all $u(v)^\omega \in E$ uniquely determine which string $s' \in S$ corresponds to the state reached in $\mathcal{M}'$ from $q$ on input symbol $\sigma$. Thus the transition graph of $\mathcal{M}'$ is isomorphic to the transition graph of $\mathcal{M}$. □

## 6 Learning the automaton

Let $L$ denote the language to be learned, and $\mathcal{M}$ denote an acceptor of $n$ states that is isomorphic to its right congruence automaton and recognizes $L$. Let the input sample of labeled examples be $T$. We now describe a learning algorithm **A** that makes use of the information in the given sample $T$ to construct an automaton. If $T$ subsumes $T_{Aut}$ the returned automaton will be isomorphic to the acceptor $\mathcal{M}$.

From the sample $T$, the algorithm constructs as follows a set $E$ of strings that serve as experiments used to distinguish states. For each labeled example $(u(v)^\omega, l)$ in $T$, all of the elements of *suffixes*$(u(v)^\omega)$ are placed in $E$. Thus if the sample $T$ includes $T_{Aut}$, then for any pair of states of $\mathcal{M}$ the set $E$ includes an experiment that distinguishes them.

Starting with the empty string $\varepsilon$, the algorithm attempts to build up a prefix-closed set $S$ of finite strings that reach different states of $\mathcal{M}$ from the initial state.

Initially, $S_1 = \{\varepsilon\}$. After $S_k$ has been constructed, the algorithm attempts to determine, for each $s \in S_k$ and each symbol $\sigma \in \Sigma$ in the ordering defined on $\Sigma$, whether $s \cdot \sigma$ reaches the same state as some string already in $S_k$ or a new state. If for each string $s'$ in $S_k$, there exists some $u(v)^\omega \in E$ such that the sample $T$ has different labels for $s \cdot \sigma \cdot u(v)^\omega$ and $s' \cdot u(v)^\omega$, then this is evidence that $s \cdot \sigma$ reaches a new state, and $S_{k+1}$ is set to $S_k \cup \{s \cdot \sigma\}$. If no such pair $s$ and $\sigma$ is found, then the final set $S$ is $S_k$. Because $\mathcal{M}$ has only $n$ states, this case is reached with $k \leq n$. If the sample $T$ subsumes $T_{Aut}$ then this process will discover exactly the strings reaching all $n$ states of $\mathcal{M}$ used in the construction of $T_{Aut}$; otherwise, it may terminate early.

In the second phase, the algorithm uses the strings in $S$ as names for states and constructs a transition function $\delta'$ using $S$ and $E$. For each $s \in S$ and $\sigma \in \Sigma$, we know that there is at least one $s' \in S$ such that there is no $u(v)^\omega \in E$ for which $s \cdot \sigma \cdot u(v)^\omega$ and $s' \cdot u(v)^\omega$ have different labels in $T$ (possibly because one or more of these examples are not in $T$ at all.) The algorithm selects one such $s'$ and defines $\delta'(s, \sigma) = s'$. If the strings in $S$ actually reach all the states of $\mathcal{M}$ and the choice of $s'$ is unique in each case, then $\delta'$ will be isomorphic to the transition function of $\mathcal{M}$. This will be the case if the sample $T$ includes $T_{Aut}$ because then among the elements of $E$ will be experiments that distinguish any pair of states of $\mathcal{M}$; otherwise, $\delta'$ may not be correct.

## 7 Characteristic sample for a DPA

The construction of $T_{Acc}$, the part of the characteristic sample used for retrieving the accepting condition of a DPA, builds on the construction of a forest of SCCs associated with a given DPA, which we term the *canonical forest*. Its properties and its construction are described next.

### 7.1 Constructing the canonical forest of a DPA

We start with some definition and simple claims.Let $\mathcal{P} = (\Sigma, Q, q_\iota, \delta, \kappa)$ be a deterministic parity acceptor (DPA). A set of states $C \subseteq Q$ is a *strongly connected component* (SCC) if and only if $C$ is nonempty and for every $q_1, q_2 \in C$, there exists a nonempty string $v \in \Sigma^+$ such that $\delta(q_1, v) = q_2$ and for all $u \preceq v$, $\delta(q_1, u) \in C$. Note that an SCC need not be maximal, and that a singleton $\{q\}$ is an SCC if and only if the state $q$ has a self-loop, that is, $\delta(q, \sigma) = q$ for some $\sigma \in \Sigma$. For any $\omega$-word $w$, the set $C$ of states visited infinitely often in the run of $\mathcal{P}$ on input $w$ is an SCC of $\mathcal{P}$.

**Claim 7.** *If $C_1$ and $C_2$ are SCCs of $\mathcal{P}$ and $C_1 \cap C_2 \neq \emptyset$, then $C_1 \cup C_2$ is also an SCC of $\mathcal{P}$.*

If $\mathcal{P}$ is a DPA and $R \subseteq Q$ is any set of states, define $SCCs(R)$ to be the set of all $C$ such that $C \subseteq R$ and $C$ is an SCC of $\mathcal{P}$. Also define $maxSCCs(R)$ to be the maximal elements of $SCCs(R)$ with respect to the subset ordering.

**Claim 8.** *If $\mathcal{P}$ is a DPA and $R \subseteq Q$ is any set of states, then the elements of maxSCCs$(R)$ are pairwise disjoint, and every set $C \in SCCs(R)$ is a subset of exactly one element of maxSCCs$(R)$.*

If $\mathcal{P}$ is a DPA, we extend its coloring function $\kappa$ to any nonempty set $R$ of states by $\kappa(R) = \min\{\kappa(q) \mid q \in R\}$. We define the *parity* of $R$ to be 1 if $\kappa(R)$ is odd, and 0 otherwise. For an $\omega$-word $w$, if the SCC $C$ is the set of states visited infinitely often in the run of $\mathcal{P}$ on $w$, then $w$ is accepted by $P$ iff the parity of $C$ is 1. Note that the union of two sets of parity $b$ is also of parity $b$. For any set of states $R \subseteq Q$, we define *minStates*$(R)$ to be the set of states $q \in R$ such that $\kappa(q) = \kappa(R)$, that is, the states of $R$ that are assigned the minimum color among all states of $R$.

**The Canonical Forest** Using these definitions we can show that there exists a forest associated with a DPA that has the following interesting properties. We provide an example for a canonical forest for a given DPA at the end of the current subsection.

**Theorem 9.** *Let $\mathcal{P} = (\Sigma, Q, q_0, \delta, \kappa)$ be a DPA. There exists a canonical forest $F^*(\mathcal{P})$ that is unique up to isomorphism and has the following properties.*

1. *There are at most $|Q|$ nodes in $F^*(\mathcal{P})$, each one a distinct SCC of $\mathcal{P}$.*
2. *The root nodes of $F^*(\mathcal{P})$ are the elements of maxSCCs$(Q)$.*
3. *The children of a node $C$ of parity $b$ are the maximal SCCs $C' \subseteq C$ of parity $1 - b$.*
4. *The children of a node $C$ are pairwise disjoint and their union is a proper subset of $C$.*
5. *For any SCC $D$ of $\mathcal{P}$, there is a unique node $C$ in $F^*(\mathcal{P})$ such that $D \subseteq C$ and $D$ is not a subset of any of the children of $C$, and $C$ and $D$ have the same parity.*

*Proof.* The root nodes of $F^*(\mathcal{P})$ are the elements of *maxSCCs*$(Q)$ and are SCCs that are pairwise disjoint, by Claim 8. Let $C$ be one of them, and assume its parity is $b$. Let $T$ be the set of SCCs that are subsets of $C$ and of parity $1 - b$. If $T = \emptyset$ then $C$ has no children and is a leaf of $F^*(\mathcal{P})$. Otherwise, the children of $C$ are the maximal elements of $T$ with respect to the subset ordering. The children of $C$ must be pairwise disjoint because if they share a state, then their union is an SCC contained in $C$ of parity $1 - b$ and is a proper superset of at least one of them, violating maximality. No child of $C$ can contain an element of *minStates*$(C)$ because otherwise the parity of the child would be $b$. Thus the union of the children of $C$ must be a proper subset of $C$. These conditions imply that there are at most $|Q|$ nodes in the forest, and that it is unique up to isomorphism.

Let $D$ be any SCC of $\mathcal{P}$. Then $D \in SCCs(Q)$, so by Claim 8, because the roots of $F^*(\mathcal{P})$ are the elements of *maxSCCs*$(Q)$, there is a unique root node $C_0$ such that $D \subseteq C_0$. Suppose the parity of $C_0$ is $b$. If $D$ is not a subset of any of the children of $C_0$, then it cannot have parity $1 - b$, so the choice $C = C_0$

satisfies the required condition. If, however, $D$ is a subset of some child $C_1$ of $C_0$, then because the children of $C_0$ are pairwise disjoint, $C_1$ is the only child of $C_0$ that contains $D$. Again, if $D$ is not a subset of any of the children of $C_1$ then $D$ and $C_1$ must have the same parity, and the choice $C = C_1$ satisfies the condition. Otherwise, we continue down the tree rooted at $C_0$ until a node $C$ is found that satisfies the condition. Note that if we arrive at a leaf $C_k$, then $D$ is not a subset of any of the children of $C_k$ (there are none) and $D$ must have the same parity as $C_k$ because otherwise $C_k$ would have at least one child. □

**The Canonical Coloring** The canonical forest $F^*(\mathcal{P})$ allows us to define a canonical coloring $\kappa^*$ for $\mathcal{P}$, as follows. The states in $(Q \setminus \bigcup maxSCCs(Q))$ are not contained in any SCC of $\mathcal{P}$ and do not affect the acceptance or rejection of any $\omega$-word. For definiteness, we assign them $\kappa^*(q) = 0$. For each node $C$ of $F^*(\mathcal{P})$, we define $\Delta(C)$ to be the set of states of $C$ that are not contained in the union of the children of $C$. For a root node $C$ of parity $b$, we define $\kappa^*(q) = b$ for all $q \in \Delta(C)$. Let $C$ be an arbitrary node of $F^*(\mathcal{P})$. If the states of $\Delta(C)$ have been assigned color $k$ by $\kappa^*$ and $D$ is a child of $C$, then the states of $\Delta(D)$ are assigned color $k+1$ by $\kappa^*$. We observe that if $q_1 \in \Delta(C)$ and $q_2$ is in a child of $C$, then $\kappa^*(q_1) < \kappa^*(q_2)$, and $\kappa^*(q_1)$ is of the same parity as $C$.

**Theorem 10.** *Let $\mathcal{P} = (\Sigma, Q, q_0, \delta, \kappa)$ be a DPA, and $\mathcal{P}'$ be $\mathcal{P}$ with the canonical coloring $\kappa^*$ for $\mathcal{P}$ in place of $\kappa$. Then $\mathcal{P}$ and $\mathcal{P}'$ recognize the same $\omega$-language.*

*Proof.* Let $w$ be an $\omega$-word and let $D$ be the SCC consisting of the states visited infinitely often in the run of $\mathcal{P}$ (and also of $\mathcal{P}'$) on input $w$. Let $C$ be the unique node of $F^*(\mathcal{P})$ such that $D$ is a subset of $C$ and is not a subset of any of the children of $C$. Thus $D$ contains at least one $q \in \Delta(C)$. In $\mathcal{P}$ the parity of $D$ is the same as the parity of $C$, which is the same as the parity of $\kappa^*(q)$, which is equal to the parity of $D$ in $\mathcal{P}'$. Thus either both $\mathcal{P}$ and $\mathcal{P}'$ accept $w$ or both reject $w$. □

**Computing the Canonical Forest** We now show that, given a DPA $\mathcal{P} = (\Sigma, Q, q_0, \delta, \kappa)$, we can compute the canonical forest of $\mathcal{P}$ in polynomial time. We first define a (possibly non-canonical) forest $F_\kappa(\mathcal{P})$ using the given coloring $\kappa$. The root nodes are the elements of $maxSCCs(Q)$, the set of all maximal SCCs of $\mathcal{P}$. Once we have defined a node $C$ of the forest, the children are the elements of the set $maxSCCs(C \setminus minStates(C))$, that is, the maximal SCCs contained in $C$ with the set of states of minimum color removed. If this set is empty, the node has no children and is a leaf. Note that in contrast to the case of the canonical forest, in $F_\kappa(\mathcal{P})$ the children of a node are not constrained to be of parity opposite to that of the parent.

By construction each node in the forest $F_\kappa(\mathcal{P})$ is an SCC of $\mathcal{P}$. If $D$ is a descendant of $C$ in the forest, then $D$ is a proper subset of $C$, and $\kappa(C) < \kappa(D)$. Because the roots are pairwise disjoint and the children of any node are pairwise disjoint, the sets $minStates(C)$ for nodes $C$ in the forest are pairwise disjoint and

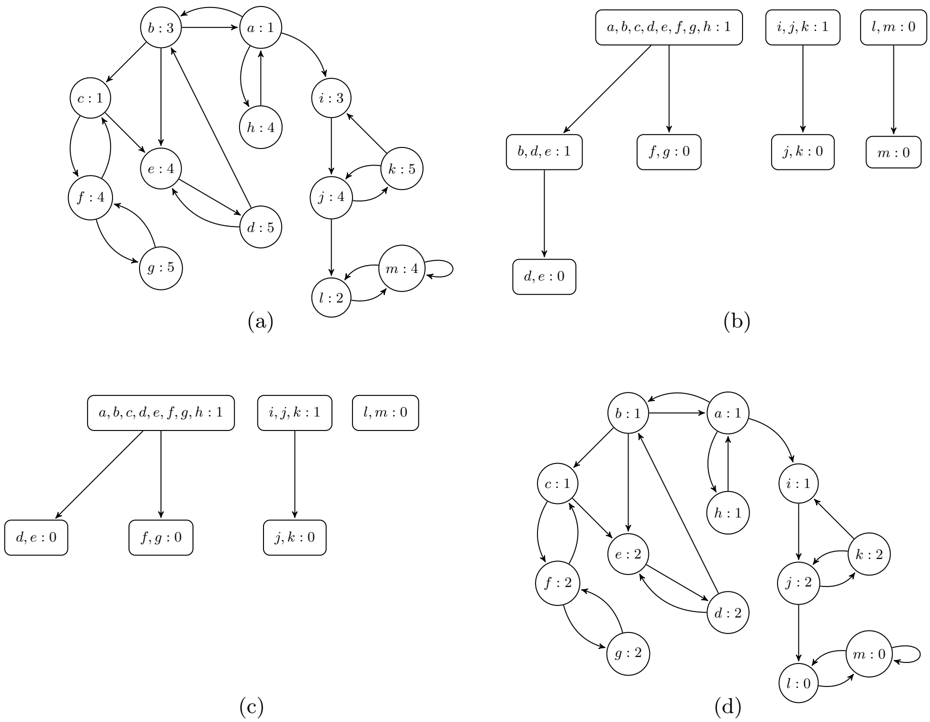

Fig. 2: (a) Transition graph of DPA $\mathcal{P}$ with states colored by $\kappa$. (b) Non-canonical forest $F_\kappa(\mathcal{P})$, with parities of nodes. (c) Canonical forest $F^*(\mathcal{P})$, with parities of nodes. (d) Transition graph of $\mathcal{P}$ with the canonical coloring $\kappa^*$.

nonempty, so there are at most $|Q|$ nodes. Because a linear time algorithm for computing strongly connected components can be used to compute the children of a node, the forest $F_\kappa(\mathcal{P})$ may be computed in polynomial time in the size of the given DPA $\mathcal{P}$.

To obtain the canonical forest $F^*(\mathcal{P})$ from the possibly non-canonical forest $F_\kappa(\mathcal{P})$, we may repeatedly merge pairs of adjacent nodes of the same parity until every pair of adjacent nodes are of different parity. That is, if $C$ is a node of parity $b$ and $D$ is a child of $C$ of parity $b$, then $D \subseteq C$, and we merge $D$ into $C$ by deleting $D$ and making all the children of $D$ direct children of $C$. Repeating this operation until there are no parent/child pairs of equal parity yields the canonical forest $F^*(\mathcal{P})$. This computation can be done in polynomial time.

Note that to obtain a canonical forest for a given DBA (resp., DCA) we can simply first color states in $F$ by 1 (resp. 0) and in $Q \setminus F$ by 2 (resp., 1) and then compute the canonical forest for the resulting DPA. In both cases the canonical forest will be of depth at most two, since in DBA an accepting SCC cannot be subsumed by a rejecting SCC (and vice versa in DCA).

*An Example* Figure 2(a) shows the transition graph of an example DPA $\mathcal{P}$ with states $a$ through $m$, labeled by the colors assigned by $\kappa$. There is a directed edge from state $q_1$ to state $q_2$ if there exists a symbol $\sigma \in \Sigma$ such that $\delta(q_1, \sigma) = q_2$.

Figure 2(b) shows the non-canonical SCC forest $F_\kappa(\mathcal{P})$ of $\mathcal{P}$, with the nodes labeled by their parities. Figure 2(c) shows the canonical SCC forest $F^*(\mathcal{P})$ of $\mathcal{P}$, with the nodes labeled by their parities. Figure 2(d) shows the transition graph of $\mathcal{P}$ re-colored using the canonical coloring $\kappa^*$.

### 7.2 Constructing the characteristic sample for a DPA

We can now construct $T_{Acc}$, the second part of the characteristic sample for a DPA $\mathcal{P}$. The sample $T_{Acc}$ consists of one example $u(v)^\omega$ for each node $C$ of the canonical forest $F^*(\mathcal{P})$, where $u$ is a string that reaches a state $q$ in $C$ from the initial state $q_0$, and $v$ is a nonempty string that, starting from $q$, visits every state of $C$ and no state outside of $C$ and returns to $q$. The length of the example $u(v)^\omega$ can be taken to be bounded by $n + n^2$. The example $u(v)^\omega$ is labeled 1 if it is accepted by $\mathcal{P}$ and otherwise is labeled 0. Then $T_{Acc}$ contains at most $n$ labeled examples, each of length polynomial in $n$. The final characteristic sample for $L = [\![\mathcal{P}]\!]$ is $T_L = T_{Aut} \cup T_{Acc}$. The sample $T_L$ contains $O(|\Sigma|n^3)$ labeled examples, each of length at most $O(n^4)$, which is polynomial in $size(L)$.

## 8 The learning algorithm for a DPA

We can now describe the learning algorithm **A** that makes use of the information in $T_L$. Similar to Gold's construction, the algorithm optimistically assumes that the sample includes a characteristic sample, and if that assumption fails to produce an acceptor consistent with the sample, the algorithm defaults to producing a table-lookup acceptor to ensure that its hypothesis is consistent with the sample. The algorithm we describe is sufficient to establish the theoretical results, but for practical applications much more effort should be expended to find good heuristic choices to avoid defaulting too easily.

Let $L$ denote the language to be learned, and $\mathcal{P}$ denote a DPA of $n$ states that is isomorphic to its right congruence automaton and recognizes $L$. The first and second phases of the algorithm are as described in Section 6: in the first phase the algorithm builds the set $S$ of states of the automaton, and in the second step it builds the transition relation $\delta'$. In the third phase, the acceptance (namely the coloring) is determined. In this phase, the algorithm may default to returning the table-lookup DPA for $T$. We first explain the construction of the table-lookup DPA then describe the third phase.

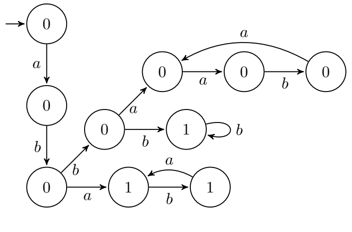

Fig. 3: Table-lookup DPA for $T = \{(a(b)^\omega, 1), ((ab)^\omega, 1), (ab(baa)^\omega, 0)\}$.

**A table-lookup DPA** A table-lookup DPA for a given sample $T$ is constructed by finding the shortest prefix of each example $u(v)^\omega$ in $T$ that distinguishes it from all other examples in $T$ and placing these prefixes in a trie-like structure. At each leaf of the trie is a structure accepting (or rejecting, depending on the label of the example) the appropriate suffix of the unique example that arrives at that leaf. By Claim 1, this DPA

can be constructed in time polynomial in the length of the sample $T$. Note that this construction is easily modified to give a DBA, DCA or DMA instead of a DPA. As an example, for the sample $T = \{(a(b)^\omega, 1), ((ab)^\omega, 1), (ab(baa)^\omega, 0)\}$, the corresponding prefixes are $abbb$, $aba$, and $abba$, and the table-lookup DPA for $T$ is shown in Figure 3, with states labeled by colors 0 and 1.

**Determining the coloring** In the third phase, the algorithm attempts to define a coloring of the states in $S$. The algorithm constructs the set $Z$ of all subsets $C$ of $S$ such that for some labeled example $(u(v)^\omega, l)$ in $T$, the subset $C$ is the set of elements of $S$ that are visited infinitely often in the run on input $u(v)^\omega$ starting at $\varepsilon$ using the transition function $\delta'$. If in this process two examples with different labels are found to yield the same set $C$, the learning algorithm defaults to the table-lookup DPA for $T$. Otherwise, each set $C$ in $Z$ is associated with the label of the example(s) that yield $C$. The set $Z$ is partially ordered by the subset relation. The learning algorithm then attempts to construct a forest $F'$ with nodes that are elements of $Z$, corresponding to the canonical forest of $\mathcal{P}$. Initially, $F'$ contains as roots all the maximal elements of $Z$. If these are not pairwise disjoint, it defaults to the table-lookup DPA for $T$. Otherwise, for each unprocessed element $C$ in $F'$, it computes the set of all $D \in Z$ such that $D \subseteq C$, $D$ has the opposite label to $C$, and $D$ is maximal with these properties, and makes $D$ a child of $C$. When all the children of a node $C$ have been determined, the algorithm checks two conditions: (1) that the children of $C$ are pairwise disjoint, and (2) there is at least one $s \in C$ that is not in any child of $C$. If either of these conditions fail, then it defaults to the table-lookup DPA for $T$. If both conditions are satisfied, then the node $C$ is marked as processed. When there are no more unprocessed nodes, the construction of $F'$ is complete. Note that $F'$ can have at most $n$ nodes, because $S$ has at most $n$ elements.

When the construction of $F'$ completes, for each node $C$ in $F'$ let $\Delta(C)$ denote the elements of $C$ that do not appear in any of its children. Then the learning algorithm assigns colors to the elements of $S$ starting from the roots of $F'$, as follows. If $C$ is a root with label $l$, then $\kappa'(s) = l$ for all $s \in \Delta(C)$. If the elements of $\Delta(C)$ have been assigned color $k$ and $D$ is a child of $C$, then $\kappa'(s) = k + 1$ for all $s \in \Delta(D)$. When this process is complete, any uncolored strings $s$ are assigned $\kappa'(s) = 0$. If the resulting DPA $\mathcal{P}'$ is consistent with the sample $T$, the learning algorithm outputs $\mathcal{P}'$ and halts. If the sample $T$ includes both $T_{Aut}$ (to specify the automaton) and $T_{Acc}$ (to specify the coloring), then $F'$ will be isomorphic to the canonical forest $F^*(\mathcal{P})$ and $\kappa'$ will correspond to the canonical coloring $\kappa^*$, and $\mathcal{P}'$ will recognize the target language $L$.

If the process described above does not result in a DPA that is consistent with the sample $T$, then the algorithm defaults to constructing the table-lookup DPA for $T$.

The learning algorithm also works for the classes $\mathbb{IB}$ and $\mathbb{IC}$: In the case of $\mathbb{IB}$ and $\mathbb{IC}$ we need to define a set $F$ rather than a coloring $\kappa$. After constructing the forest, the set $F$ is determined to contain the states in the root nodes that are not in the leaves. Thus we have the following.

**Theorem 11.** *The classes $\mathbb{IB}$, $\mathbb{IC}$ and $\mathbb{IP}$ are identifiable in the limit using polynomial time and data. Moreover, characteristic samples can be computed in polynomial time.*

A corollary of Theorem 11 is that the class of languages recognized by derministic weak parity acceptors ($\mathbb{DWPA}$) which was shown to be polynomially learnable using membership and equivalence queries in [24] is identified in the limit using polynomial time and data. This class (which is equivalent to the intersection of classes $\mathbb{DBA} \cap \mathbb{DCA}$) was shown to be a subset of $\mathbb{IM}$ in [30], and to be a subset of $\mathbb{IP}$ in [4].

**Corollary 2.** *The class $\mathbb{DWPA}$ is identifiable in the limit using polynomial time and data. Moreover, characteristic samples can be computed in polynomial time.*

## 9 The sample $T_{Acc}$ and the learning algorithm for a DMA

The above results can be extended to the class $\mathbb{IM}$. Recall that we define the size measure for a DMA to be $\max\{|\Sigma|, |Q|, m\}$, where $m$ is the number of sets in the acceptance condition. For the characteristic sample $T_L$, $T_{Aut}$ remains the same, but $T_{Acc}$ contains for each accepting set $C$, an example $u(v)^\omega$ for which $C$ is the set of states visited infinitely often. In the learning algorithm, the construction of the transition function remains the same. Instead of attempting to construct a coloring function, the learning algorithm finds for each labeled example $(u(v)^\omega, 1) \in T$, the set $C$ of states $s$ that are visited infinitely often on input $u(v)^\omega$ starting from $\varepsilon$ and using the transition function $\delta'$, and adds $C$ to the acceptance condition. If the construction does not result in a DMA consistent with $T$, then it defaults to producing a table-lookup DMA for $T$. Because in addition, as stated in Section 5.1, a characteristic samples can be computed in polynomial time, we have the following.

**Theorem 12.** *The class $\mathbb{IM}$ is identifiable in the limit using polynomial time and data. Moreover, a characteristic sample can be computed in polynomial time.*

## 10 Discussion

We have shown that the non-deterministic classes of $\omega$-automata $\mathbb{NBA}$, $\mathbb{NPA}$, $\mathbb{NMA}$ and $\mathbb{NCA}$ cannot be identified in the limit using polynomial data. A negative result regarding query learning of the first three classes was recently obtained in [3]. That result makes a plausible assumption of cryptographic hardness, which is not required here. On the positive side we have shown that the classes $\mathbb{IB}$, $\mathbb{IC}$, $\mathbb{IP}$ and $\mathbb{IM}$ can be identified in the limit using polynomial time and data. And moreover, a characteristic sample can be constructed in polynomial time. The construction builds on the definition of a canonical forest for a DPA which may be of use in other contexts as well. The question whether the deterministic classes $\mathbb{DBA}$, $\mathbb{DPA}$, $\mathbb{DMA}$ and $\mathbb{DCA}$ can be polynomially learned in the limit remains open.
